# Complexities in species identification for *Serratia marcescens* complex for the modern microbiology laboratory

**DOI:** 10.1128/spectrum.01361-24

**Published:** 2024-12-09

**Authors:** Susan A. J. Harch, Frances Jenkins, Rima Farhat, Sebastiaan J. van Hal

**Affiliations:** 1 Department of Microbiology and Infectious Diseases, Royal Prince Alfred Hospital, Sydney, Australia; University of California San Diego, La Jolla, California, USA

**Keywords:** *S. marcescens*, mass spectrometry, whole-genome sequencing, species identification

## LETTER

The Gram-negative bacterium *Serratia marcescens* is a major nosocomial pathogen and is increasingly associated with healthcare outbreaks (1, 2). Accurate species identification is fundamental to optimal microbiology reporting, as species may differ in environmental niches, clinical syndromes, and carriage of antimicrobial resistance and virulence genes. As such, identification at a species level frequently informs outbreak investigation, clinical management, and epidemiological studies (1, 3).

Matrix-assisted laser desorption/ionization time-of-flight mass spectrometry (MALDI-TOF MS) is frequently used for pathogen identification; however, species discrimination for some bacteria is limited by a high degree of similarity between mass spectra. Although whole-genome sequencing (WGS) can provide improved taxonomic granularity, this depends on the depth and accuracy of pathogen sequences within the databases. Studies of the population structure of *S. marcescens* are few (1, 3, 4) with none correlating MALDI-TOF MS with WGS.

We compared species identification of 19 purported *S. marcescens* isolates (suspected nosocomial outbreak, *n* = 10) by MALDI-TOF MS with WGS using two separate tools, Kraken2 (5) and Ribosomal Multi-locus Sequence Typing (rMLST) (6).

Following 24 hours incubation at 37°C in 5% CO_2_ on horse blood agar (ThermoFisher Scientific, Australia), isolates were identified by mass spectrometry using the Bruker MALDI Biotyper IVD, v.4.1.100. For WGS, DNA from a single colony was extracted using EZ1 DSP Virus Kit, (Qiagen, Germany), library generation by Illumina DNA Prep Kit (Illumina, USA), and sequencing on the Illumina iSeq100.

Raw reads were trimmed using fastp (v.0.23.4) prior to species identification with Kraken2 (v.2.1.3), a tool that uses a *k*-mer-based approach with short genomic strings mapped to the lowest common taxonomic ancestor within the Kraken2 database (5). In addition, *de novo* assemblies were constructed using SPAdes (v.3.15.5) with rMLST profiles determined by indexing the genomic diversity across 53 ribosome protein subunits (*rps*) genes obtained from PubMLST (6, 7).

Using the MALDI-TOF MS, isolates were reported as *S. marcescens* (*n* = 15) or *Serratia nematodiphila* (*n* = 2) with a “high-confidence identification” score (>2.0) (Table 1). Using Kraken2, the highest-ranked species for all isolates was *S. marcescens*. In contrast, rMLST demonstrated considerable taxonomic diversity. Five species were represented, each with robust allele support (>90% representative of high confidence) (8).

**TABLE 1:** Comparative species identification by MALDI-TOF MS and WGS

Isolate	Sample	MALDI-TOF MS	Kraken2 (WGS)		Ribosomal MLST (WGS)	
		Best organism match (score)	% classified reads mapping to *Serratia* genus	Highest rank species	rMLST species identification	Allele support (%)
1	Environmental	*S. marcescens* (1.96)	83.96	*S. marcescens*	*S. nevei*	100
2	Environmental	*S. marcescens* (2.07)	81.26	*S. marcescens*	*S. nevei*	100
3	Environmental	*S. marcescens* (2.22)	87.09	*S. marcescens*	*S. nevei*	96
4	Environmental	*S. marcescens* (2.25)	93.81	*S. marcescens*	*S. nevei*	96
5	Environmental	*S. marcescens* (2.25)	80.97	*S. marcescens*	*S. nevei*	100
6	Eye swab	*S. marcescens* (2.19)	95.01	*S. marcescens*	*S. nevei*	96
7	Eye swab	*S. marcescens* (1.97)	94.90	*S. marcescens*	*S. nevei*	96
8	Eye swab	*S. marcescens* (2.27)	95.02	*S. marcescens*	*S. nevei*	96
9	Eye swab	*S. marcescens* (2.4)	94.94	*S. marcescens*	*S. marcescens*	87
10	Rectal swab	*S. nematodiphila* (2.36)	94.96	*S. marcescens*	*S. marcescens*	87
11	Blood culture	*S. marcescens* (2.44)	93.58	*S. marcescens*	*S. marcescens*	100
12	Blood culture	*S. marcescens* (2.4)	95.46	*S. marcescens*	*S. bockelmannii*	98
13	Blood culture	*S. marcescens* (2.2)	90.78	*S. marcescens*	*S. nematodiphila*	98
14	Blood culture	*S. marcescens* (2.08)	94.92	*S. marcescens*	*S. nevei*	100
15	Blood culture	*S. marcescens* (2.19)	93.54	*S. marcescens*	*S. ureilytica*	100
16	Sputum	*S. marcescens* (2.32)	90.65	*S. marcescens*	*S. nematodiphila*	98
17	Sputum	*S. nematodiphila* (2.32)	95.14	*S. marcescens*	*S. nevei*	96
18	Urine	*S. marcescens* (2.27)	95.50	*S. marcescens*	*S. ureilytica*	100
19	Colostomy screen	*S. marcescens* (2.29)	95.62	*S. marcescens*	*S. bockelmannii*	89

The Bruker “Identification Results” include the footnote “Species *marcescens/nematophila/ureilytica* of the genus *Serratia* have very similar patterns: Therefore, distinguishing their species is difficult.” The MBT database also does not include *Serratia bockelmannii* and *Serratia nevei* (9). Reporting as “*S. marcescens* complex*”* by MALDI-TOF is most accurate; however, to date, a formal taxonomic classification of *Serratia marcescens* complex (SMC) has not been established by the National Centre for Biotechnology Information. Recent genomic studies have proposed a classification of SMC including *S. nematodiphila, S. marcescens, S. bockelmannii, S. ureilytica*, and *S. nevei* (3), with uncertainty regarding the inclusion of *Serratia surfactantfaciens* (1). These findings support the prior call to “revise the taxonomic classification of SMC” (3).

The Kraken2 database includes all the above species except *S. bockelmannii*; yet it was unable to differentiate the respective species despite using the most up-to-date standard database (updated 12/1/2024, https://benlangmead.github.io/aws-indexes/k2). We hypothesize that there are insufficient unique kmers to accurately assign a species due to the high nucleotide-level genomic similarity within the *S. marcescens* complex (3).

In contrast, the use of conserved genomic regions likely explains why the rMLST method was able to obtain a species-level identification in most isolates. We note that this differs from Aracil-Gisbert et al. (3) and suggest that this is due to the continued expansion of tool databases.

In conclusion, MALDI-TOF MS is unable to reliably differentiate *S. marcescens sensu stricto* from genomically similar subspecies, and reporting should be as *S. marcescens* complex, as suggested above (3). WGS can further define species identification but it is essential to be aware of tool limitations with organisms of a low genomic diversity.

## Data Availability

Trimmed reads were submitted to the National Center for Biotechnology Information (NCBI) sequence read archive under project accession number PRJNA1177597.
